# Tigecycline versus doxycycline for scrub typhus: a preliminary randomized trial

**DOI:** 10.1186/s41182-026-01073-8

**Published:** 2026-09-16

**Authors:** Jae Hyoung Im, Sukbin Jang, Young Ju Suh, DaeHyung Lee, Kwang Jun Lee, Jonghyun Kim, Se Ju Lee, Ji Hyeon Baek, Young Kyoung Park, EunJi Kim, Sun-Myoung Lee, Hye-Jin Lee, Moon-Hyun Chung, Kyung-Wook Hong, In-Gyu Bae, Jin-Soo Lee

**Affiliations:** 1 Division of Infectious Diseases, Department of Internal Medicine, Inha University College of Medicine, Incheon, Republic of Korea; 2 Division of Infectious Diseases, Department of Internal Medicine, Dankook University College of Medicine, Cheonan, Republic of Korea; 3 The Biostatistics Center, Biomedical Research Institute, Inha University College of Medicine, Incheon, Republic of Korea; 4 Division of Zoonotic Infectious Diseases, Korea Disease Control and Prevention Agency, Osong, Republic of Korea; 5 Translational Research Center, Institute for Bio-Medical and Translational Health Care, Inha University College of Medicine, Incheon, Republic of Korea; 6 Division of Infectious Diseases, Department of Internal Medicine, Gyeongsang National University College of Medicine, Jinju, Republic of Korea

**Keywords:** Scrub typhus, *Orientia tsutsugamushi*, Tigecycline, Doxycycline, Randomized trial

## Abstract

**Background:**

Doxycycline is the standard treatment for scrub typhus, but evidence regarding parenteral alternatives remains limited in hospitalized patients.

**Main body:**

We conducted a multicenter, prospective, randomized, open-label trial in Korea. Participants were enrolled and randomized from November 2022 through December 6, 2024. Adults aged 19 years or older hospitalized with clinically suspected scrub typhus were randomized to intravenous tigecycline or oral doxycycline; all randomized participants were subsequently laboratory-confirmed by PCR and/or seroconversion on paired serologic testing. Tigecycline was given as a 100 mg loading dose followed by 50 mg every 12 h for 5 days; doxycycline was given as 100 mg every 12 h for 7 days. The primary outcome was time from first dose to body temperature <37.3 °C sustained for at least 48 consecutive hours without antipyretics. Of 27 randomized patients, 24 were included in the per-protocol efficacy analysis. Median time to defervescence was shorter with tigecycline than with doxycycline (16.6 h [interquartile range, 11.0–32.0] vs. 55.0 h [24.0–64.3]; *P* = 0.0079). No recurrence requiring additional antirickettsial therapy occurred within 30 days. One patient in each group reported nausea.

**Conclusions:**

In this preliminary randomized trial, intravenous tigecycline was associated with faster defervescence than oral doxycycline in clinically stable hospitalized adults with scrub typhus. These findings support further evaluation of tigecycline as an intravenous option in selected patients.

*Trial registration* ClinicalTrials.gov, NCT07513103.

**Supplementary Information:**

The online version contains supplementary material available at https://doi.org/10.1186/s41182-026-01073-8.

## Background

Scrub typhus is an acute febrile illness caused by *Orientia tsutsugamushi*, an obligate intracellular gram-negative bacterium. Oral doxycycline remains the standard treatment for most patients [1, 2]. However, an intravenous option may be useful in selected hospitalized patients with persistent vomiting, impaired swallowing, altered mental status, or uncertain gastrointestinal absorption. Evidence for alternatives is limited. Macrolides and other agents have been studied in mild-to-severe scrub typhus, but comparative data on intravenous treatment strategies remain sparse [3, 4]. Tigecycline has in vitro activity against *O. tsutsugamushi* and achieves intracellular concentrations, providing a biologically plausible rationale for clinical evaluation [5, 6]. This preliminary randomized trial compared early clinical response between intravenous tigecycline and oral doxycycline in adults hospitalized with scrub typhus.

Because *O. tsutsugamushi* is intracellular, early clinical response may depend not only on in vitro susceptibility but also on intracellular drug exposure, route of administration, and the timing of effective concentrations after treatment initiation. Most prior comparative trials have focused on oral regimens or on severe scrub typhus treatment strategies. Therefore, the present study was designed as an early signal-seeking trial in clinically stable hospitalized adults who could receive oral doxycycline if randomized, rather than as a trial specifically limited to patients with unreliable oral administration. The present study was designed to address this narrow clinical question rather than to establish a new universal first-line regimen.

## Main text

### Study design and participants

This multicenter, prospective, randomized, open-label trial was conducted at Inha University Hospital, Dankook University Hospital, and Gyeongsang National University Hospital in Korea. The original protocol was approved by the Institutional Review Board of Inha University Hospital on June 13, 2022, and the study underwent the required regulatory review and reporting procedures in Korea before participant enrollment. Screening began in October 2022, and the first participant was enrolled and randomized on November 19, 2022. A protocol amendment revising the primary-outcome definition was approved on March 27, 2023. The study information was subsequently submitted to ClinicalTrials.gov (NCT07513103) on August 16, 2023. The delay in ClinicalTrials.gov registration resulted from an administrative delay in completing the registry entry and did not reflect a delay in institutional or regulatory approval of the trial. Enrollment of the remaining study cohort continued through December 6, 2024, and follow-up of the last participant was completed on January 5, 2025. Adults aged 19 years or older who were hospitalized with clinically suspected scrub typhus were screened and could be enrolled before laboratory confirmation. Because participants randomized to the control group received oral doxycycline, eligibility was restricted to patients who were able to take oral medication at enrollment and had no clinical condition that precluded oral administration. Scrub typhus was confirmed by paired serology or polymerase chain reaction (PCR). Serologic confirmation was defined as seroconversion on paired indirect immunofluorescence assay samples, and PCR positivity was defined as detection of the 56-kDa antigen gene of *O. tsutsugamushi* by nested PCR in whole blood [7]. All randomized participants were ultimately laboratory-confirmed, and no participant was excluded because scrub typhus was not confirmed. Patients were excluded if they had acute respiratory distress syndrome, shock, encephalopathy, or significant immunosuppression at screening, because the trial was designed to assess early response in clinically stable hospitalized adults before evaluation in more severe disease.

Screening was performed irrespective of current fever status to allow rapid study consideration after diagnostic suspicion or confirmation. However, patients who were afebrile at formal eligibility assessment were not randomized, because the primary outcome required an interpretable febrile starting point and fever clearance is most informative when measured from active febrile illness.

This preliminary randomized trial was designed to estimate an early clinical efficacy signal and its uncertainty rather than to provide definitive evidence of treatment superiority. No formal hypothesis-testing power calculation was performed. The planned target sample size was 40 patients, with 20 patients in each treatment group, as a pragmatic sample size for this preliminary randomized trial; however, 27 patients were randomized because eligible patient enrollment was limited during the study period, and 24 were included in the per-protocol efficacy analysis.

### Interventions and outcomes

Eligible participants were assigned in a 1:1 ratio through centralized allocation according to a computer-generated randomization sequence prepared by an independent statistician. Allocation was concealed from investigators until assignment. Because the study compared intravenous and oral regimens, the trial was open-label. Patients assigned to tigecycline received a 100 mg intravenous loading dose followed by 50 mg intravenously every 12 h for 5 days. Patients assigned to doxycycline received oral doxycycline 100 mg every 12 h for 7 days. Medication histories and medical records also confirmed that no participant had received systemic antibiotics before study enrollment, including antirickettsial agents, cefotaxime, or other beta-lactam antibiotics. The primary outcome was time to defervescence, defined as the interval from the first dose of study antibiotic to body temperature <37.3 °C sustained for at least 48 consecutive hours without antipyretics. This definition was approved in a protocol amendment on March 27, 2023, after enrollment of the first participant but before enrollment of the remaining study cohort and before any comparative analysis of treatment-group outcomes. The amended definition was subsequently included in the ClinicalTrials.gov record submitted on August 16, 2023. The original protocol definition of body temperature <37.5 °C sustained for at least 48 h was evaluated in a sensitivity analysis. Body temperature was measured every 4 h at the tympanic membrane using infrared tympanic thermometers. The same anatomical site and measurement method were used at all participating centers and were maintained consistently within each participant, although the manufacturer and model of the thermometer were not mandated as a single device across centers. Among the 24 participants included in the per-protocol efficacy analysis, no temperature measurements required for primary-outcome determination were missing. Time to defervescence was calculated using the actual time of the first study-antibiotic dose and the actual time of the first qualifying temperature measurement. Minute-level intervals were converted to decimal hours by dividing the number of minutes by 60. All medications administered after the first study-antibiotic dose were reviewed for potential antipyretic activity. Acetaminophen and propacetamol were classified as potential antipyretics, whereas tramadol was not. For each administration, the drug, dose, elapsed time from study-treatment initiation, and its temporal relationship to the recorded onset of defervescence were reviewed. Patient-level data are provided in Supplementary Table S2. The primary outcome was based on prespecified temperature measurements rather than subjective symptom assessment. Secondary outcomes included defervescence by day 3, recurrence within 30 days, and adverse events.

### Statistical analysis

The primary efficacy analysis was performed in the per-protocol population, defined as randomized participants who received the allocated study treatment, had evaluable temperature data for assessment of the primary endpoint, and had no major protocol deviations affecting endpoint assessment. This population was selected because the primary endpoint, time to sustained defervescence, required complete and interpretable temperature data. Adverse events were summarized descriptively in the per-protocol efficacy population. Continuous variables were summarized as medians with interquartile ranges (IQRs) or means with standard deviations, as appropriate, and categorical variables as numbers and percentages. Time to defervescence was compared using the Mann–Whitney *U* test. The between-group location shift for the primary endpoint was estimated using the Hodges–Lehmann estimator with a 95% confidence interval. Kaplan–Meier curves and the log-rank test were used as supportive time-to-event analyses. A two-sided *P* value <0.05 was considered statistically significant.

## Results

Of 31 screened patients, 4 were excluded before randomization because they were afebrile at formal eligibility assessment. A total of 27 patients underwent randomization. Three randomized participants were not included in the per-protocol efficacy analysis because the primary endpoint could not be validly assessed: two in the doxycycline group withdrew consent before completion of endpoint assessment, and one in the tigecycline group discontinued participation before completion of endpoint assessment because of an event unrelated to the study medication. Therefore, 24 participants were included in the per-protocol efficacy analysis (12 in each group; Additional file 1: Figure S1). All randomized participants were subsequently laboratory-confirmed by PCR and/or seroconversion on paired serologic testing; no participant was excluded because scrub typhus was not laboratory-confirmed. Baseline characteristics, including age, sex, symptom duration, presenting features, and key laboratory findings, were generally comparable between groups (Additional file 1: Table S1). Among participants included in the per-protocol efficacy analysis, no additional major protocol deviations occurred that affected assessment of sustained defervescence.

**Fig. 1:**
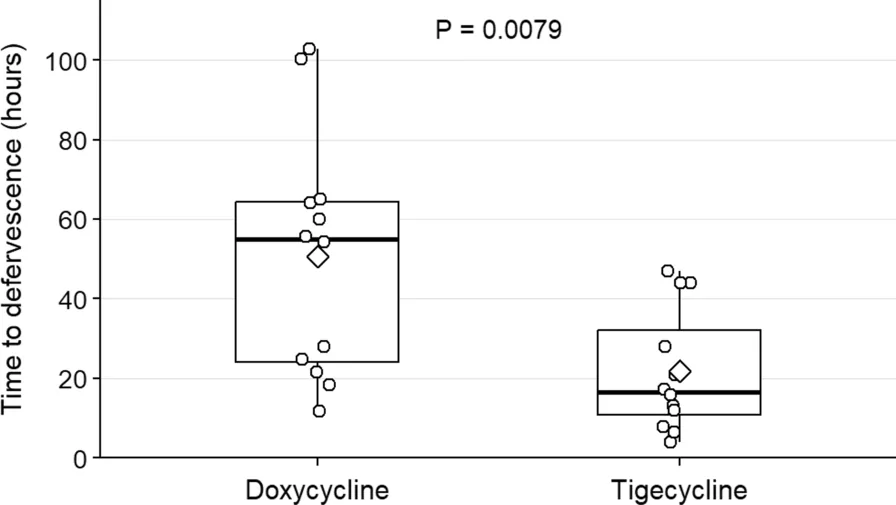
Time to defervescence by treatment group. Boxplots show time from first antibiotic dose to body temperature <37.3 °C sustained for at least 48 consecutive hours without antipyretics. Individual patient values are overlaid; diamonds indicate means. The median time to defervescence was 16.6 h (IQR, 11.0–32.0) with tigecycline and 55.0 h (IQR, 24.0–64.3) with doxycycline (*P* = 0.0079 by Mann–Whitney *U* test)

Using the amended primary-outcome definition of body temperature <37.3 °C sustained for at least 48 consecutive hours without antipyretics, the median time to defervescence was 16.6 h (IQR, 11.0–32.0) in the tigecycline group and 55.0 h (IQR, 24.0–64.3) in the doxycycline group (P = 0.0079; Fig. 1). The Hodges–Lehmann estimate of the location shift, calculated as tigecycline minus doxycycline, was −21.0 h (95% CI −50.8 to −7.2). By day 3, defervescence was achieved in all 12 participants (100%) in the tigecycline group and 10 of 12 participants (83.3%) in the doxycycline group (Additional file 1: Figure S2).

In a sensitivity analysis using the original protocol threshold of <37.5 °C sustained for at least 48 h, the median time to defervescence was 10.4 h (IQR, 7.5–24.3) in the tigecycline group and 38.9 h (IQR, 18.9–53.2) in the doxycycline group (*P* = 0.0079). The corresponding Hodges–Lehmann estimate was −20.1 h (95% CI −41.5 to −5.1), demonstrating consistency with the primary analysis.

No recurrence requiring additional antirickettsial treatment occurred within 30 days. One patient in each group reported nausea; no treatment discontinuation or serious adverse event was attributed to study medication (Additional file 1: Table S2). During the study observation period, no patient in either treatment group developed new or progressive organ dysfunction, required vasopressor therapy, renal replacement therapy, mechanical ventilation, or intensive care unit admission, or died.

## Discussion

The findings should be interpreted as an early efficacy signal rather than evidence to replace doxycycline as first-line therapy. Important comparators not evaluated in the present study include intravenous doxycycline and azithromycin. However, the rationale for evaluating tigecycline was not merely its availability as an intravenous formulation. Several factors may explain the shorter defervescence observed with tigecycline. Intravenous administration and a loading dose may have enabled rapid attainment of therapeutic exposure, and intracellular penetration may be relevant for *O. tsutsugamushi* infection [5, 6]. In addition, tigecycline has important limitations, including higher cost, gastrointestinal adverse events, potential hepatic toxicity, and antimicrobial stewardship concerns because it is generally considered a reserve agent for multidrug-resistant bacterial infections; therefore, its role should be interpreted only in selected clinical situations [8].

In previous studies, time to defervescence with doxycycline varied from 12 to 96 h [9, 10], which was broadly consistent with the range observed in the present study. Moreover, the direction and approximate magnitude of the treatment effect were consistent using both the amended <37.3 °C definition and the original <37.5 °C threshold, suggesting that the main finding was not dependent on selection of a particular temperature cutoff. Taken together, the magnitude and consistency of the difference in early fever clearance are noteworthy for hypothesis generation. However, the Hodges–Lehmann estimate had a wide confidence interval, reflecting substantial uncertainty arising from the small sample. Accordingly, the observed effect size should be interpreted as a preliminary and imprecise estimate rather than a definitive estimate of treatment efficacy. Any future sample-size calculation based on this result should use conservative assumptions and account for this uncertainty.

Defervescence is clinically relevant but does not capture the full spectrum of outcomes. Although faster defervescence may suggest earlier antibacterial activity, it does not necessarily imply reductions in organ dysfunction, length of hospital stay, need for intensive care, or mortality. In the present study, clinically meaningful outcome events, including death or organ failure, did not occur in either group, likely because the study population consisted of clinically stable patients and patients with severe scrub typhus were excluded. However, this study was small, open-label, and restricted to clinically stable hospitalized patients. These results should therefore not be extrapolated to severe scrub typhus, critically ill patients, or substantially immunosuppressed populations.

Although defervescence was achieved by day 3 in all participants in the tigecycline group and 10 of 12 participants in the doxycycline group, and no recurrence was observed within 30 days, the trial was not powered to compare relapse, complications, hospitalization duration, or mortality. Baseline ALT levels were higher in the doxycycline group; therefore, residual confounding related to hepatic involvement cannot be completely excluded.

Future studies should prespecify clinically important differences in fever clearance, include more severe disease strata, and evaluate whether faster defervescence translates into patient-centered outcomes or improved feasibility of care.

## Conclusions

This preliminary randomized trial showed an early efficacy signal of faster defervescence with intravenous tigecycline in clinically stable hospitalized patients with scrub typhus, supporting further evaluation of tigecycline as a potential parenteral option in selected clinical settings.

## Supplementary Information

Additional File 1. DOCX file containing Figure S1 (flowchart of patient enrollment and analysis), Figure S2 (Kaplan–Meier curve for time to defervescence), Figure S3 (sensitivity analysis using the original <37.5°C threshold), Table S1 (baseline characteristics), and Table S2 (individual patient data for time to defervescence, recurrence within 30 days, and adverse events).

## Data Availability

The de-identified participant-level data supporting the findings of this study are not publicly available due to patient privacy and institutional review board restrictions. Data are available from the corresponding author upon reasonable request and with appropriate institutional approval.

## Abbreviations

IQR: Interquartile range
IRB: Institutional review board
PCR: Polymerase chain reaction
