# Supplementary material for: Tigecycline versus doxycycline for scrub typhus: a preliminary randomized trial
Source: Trop Med Health. 2026 Sep 16;54:200. doi: 10.1186/s41182-026-01073-8 (PMC13579949; doi:10.1186/s41182-026-01073-8)
Supplement: Supplementary file 1 [file 41182_2026_1073_MOESM1_ESM.docx]

**Additional file 1**

**Supplementary materials for: Tigecycline versus Doxycycline for Scrub Typhus: A Preliminary Randomized Trial**

**Supplementary Figure S1. Flowchart of patient enrollment and analysis**

Flow diagram illustrating patient screening, randomization, and inclusion in the per-protocol efficacy analysis. Screening was performed irrespective of current fever status to allow rapid consideration for study participation after scrub typhus was suspected or confirmed. Patients who were afebrile at the time of formal eligibility assessment were excluded before randomization because the primary outcome required an interpretable febrile starting point. Among 27 randomized patients, three were excluded from the per-protocol efficacy analysis because the primary endpoint could not be validly assessed: two withdrew consent before completion of endpoint assessment, and one discontinued participation before completion of endpoint assessment because of an event unrelated to the study medication.

**Supplementary Table S1. Baseline characteristics by treatment group**

| **Variable** | **Doxycycline (N=12)** | **Tigecycline (N=12)** | **P value** |
| --- | --- | --- | --- |
| Maximum body temperature, °C | 38.8 ± 0.6 | 38.8 ± 0.5 | 0.941 |
| Male sex | 6 (50.0) | 3 (25.0) | 0.399 |
| Age, years | 68.5 ± 11.7 | 67.6 ± 10.5 | 0.842 |
| Hypertension | 6 (50.0) | 4 (33.3) | 0.679 |
| Diabetes mellitus | 1 (8.3) | 2 (16.7) | 1.000 |
| Dyslipidemia | 3 (25.0) | 1 (8.3) | 0.584 |
| Cardiovascular disease | 1 (8.3) | 1 (8.3) | 1.000 |
| Malignancy | 1 (8.3) | 1 (8.3) | 1.000 |
| Duration of fever before hospitalization, days | 4.7 ± 5.1 | 6.2 ± 4.5 | 0.448 |
| Eschar | 11 (91.7) | 11 (91.7) | 1.000 |
| White blood cell count, /µL | 9770.0 ± 4110.1 | 8744.2 ± 2600.7 | 0.473 |
| Platelet count, ×10³/µL | 187.4 ± 67.8 | 148.3 ± 45.3 | 0.110 |
| Alanine aminotransferase, IU/L | 126.6 ± 66.9 | 74.4 ± 47.4 | 0.038* |
| Total bilirubin, mg/dL | 0.7 ± 0.3 | 0.7 ± 0.4 | 0.703 |
| Blood urea nitrogen, mg/dL | 15.9 ± 6.4 | 18.3 ± 10.7 | 0.511 |
| Creatinine, mg/dL | 1.0 ± 0.3 | 1.0 ± 0.3 | 0.875 |
| C-reactive protein, mg/dL | 10.36 ± 5.91 | 10.14 ± 5.07 | 0.921 |
| SOFA score | 1.0 (0.0–1.0) | 1.0 (0.0–1.25) | 0.537 |
| O. tsutsugamushi PCR positive | 8 (66.7) | 9 (75.0) | 1.000 |
| O. tsutsugamushi IgM (≥1:10) | 12 (100.0) | 11 (91.7) | 1.000 |
| O. tsutsugamushi IgG (≥1:128) | 11 (91.7) | 11 (91.7) | 1.000 |
| O. tsutsugamushi IgG (≥1:256) | 4 (33.3) | 2 (16.7) | 0.640 |
| O. tsutsugamushi IgG (≥1:512) | 3 (25.0) | 1 (8.3) | 0.590 |

Values are presented as mean ± standard deviation, median (interquartile range), or n (%). An asterisk (*) indicates P < 0.05.

**Supplementary Table S2. Individual patient data**

| **No.** | **Treatment group** | **Time to defervescence, <37.5°C (h)** | **Time to defervescence, <37.3°C (h)** | **Antipyretic after study initiation (drug, dose, timing)** | **Recurrence within 30 days** | **Adverse event** |
| --- | --- | --- | --- | --- | --- | --- |
| 1 | Doxycycline | 28.0 | 28.0 | Acetaminophen 650 mg (+18.9 h) | No | None |
| 2 | Doxycycline | 60.0 | 60.0 | None | No | None |
| 3 | Doxycycline | 50.7 | 55.7 | Propacetamol 1 g (+20.9 h) | No | None |
| 4 | Tigecycline | 4.0 | 4.0 | None | No | None |
| 5 | Tigecycline | 44.0 | 44.0 | None | No | None |
| 6 | Tigecycline | 23.0 | 47.0 | None | No | None |
| 7 | Doxycycline | 64.0 | 64.0 | None | No | None |
| 8 | Tigecycline | 20.9 | 20.9 | None | No | Nausea |
| 9 | Doxycycline | 11.7 | 11.7 | None | No | None |
| 10 | Tigecycline | 40.0 | 44.0 | Acetaminophen 1 g (+2.3 h) | No | None |
| 11 | Tigecycline | 7.8 | 7.8 | None | No | None |
| 12 | Doxycycline | 12.8 | 24.8 | None | No | None |
| 13 | Doxycycline | 19.6 | 21.6 | Propacetamol 1 g (+3.8 h and + 10.8h) | No | Nausea |
| 14 | Doxycycline | 16.9 | 18.3 | None | No | None |
| 15 | Tigecycline | 8.6 | 13.2 | None | No | None |
| 16 | Tigecycline | 5.2 | 17.3 | None | No | None |
| 17 | Tigecycline | 6.6 | 6.6 | None | No | None |
| 18 | Doxycycline | 31.0 | 102.7 | None | No | None |
| 19 | Doxycycline | 65.0 | 65.0 | None | No | None |
| 20 | Tigecycline | 8.0 | 12.0 | None | No | None |
| 21 | Tigecycline | 12.2 | 15.9 | None | No | None |
| 22 | Doxycycline | 46.7 | 100.3 | None | No | None |
| 23 | Doxycycline | 50.9 | 54.2 | None | No | None |
| 24 | Tigecycline | 28.0 | 28.0 | None | No | None |

Time to defervescence was calculated from the first study-antibiotic dose to the onset of body temperature <37.3°C sustained for at least 48 consecutive hours without antipyretics. The original <37.5°C threshold sustained for at least 48 hours was evaluated as a sensitivity analysis.

**Supplementary Figure S2. Kaplan–Meier curves for time to defervescence using the amended primary-outcome definition.**

Defervescence was defined as body temperature <37.3°C sustained for at least 48 consecutive hours without antipyretics. The solid line represents doxycycline and the dotted line represents tigecycline. The log-rank P value was 0.0022.

**Supplementary Figure S3. Time to defervescence by treatment group.**

Boxplots show time from first antibiotic dose to body temperature <37.5°C sustained for at least 48 h.
